# Translational web robots for pathogen genome analysis

**DOI:** 10.1186/2042-5783-1-10

**Published:** 2011-10-31

**Authors:** Vitali Sintchenko, Enrico W Coiera

**Affiliations:** 1 Centre for Infectious Diseases and Microbiology-Public Health, Institute of Clinical Pathology and Medical Research, Westmead Hospital, Sydney, New South Wales, 2145 Australia; 2 Sydney Emerging Infections and Biosecurity Institute and Sydney Medical School-Westmead, University of Sydney, Sydney, New South Wales, 2006 Australia; 3 Centre for Health Informatics, Australian Institute of Health Innovation, University of New South Wales, Sydney, New South Wales, 2052 Australia

## 

In this letter, we present the concept of translational computational web robots that aims to improve the clinical knowledge utilization and microbiology test interpretation. This development promise to accelerate discovery by searching and will be challenging the position of observation and experimentation as the most productive means of discovery.

**Figure 1:**
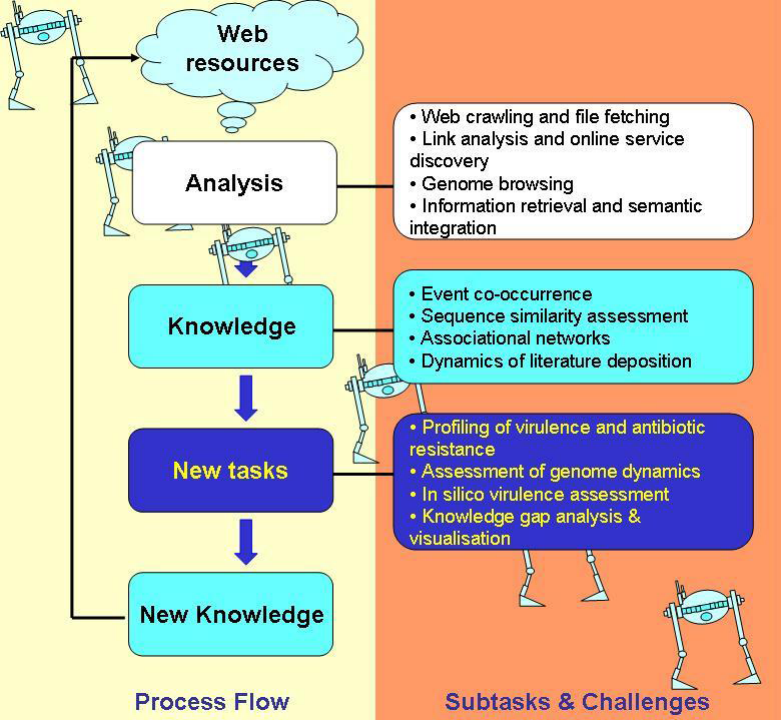
**Process flow employed by translational robots and subtasks of relevance to the work of a microbiologist**.

With a shift in the 'wet lab' bottleneck from data generation to data analysis, there has been a reinvigoration of the rusty partnership between biomedical and computer scientists. One of the most appealing targets for collaboration has been whole genome analysis, especially the linkage of new sequencing data with the wealth of unstructured information captured in biomedical literature. This endeavour holds particular promise for translational medical microbiology, where a potentially unlimited number of sequenced microbial genomes might be coupled with the breadth of infectious diseases literature. However, generation of meaningful associations between literature and sequences demands novel analytical approaches. We argue that web robots, initially called 'intelligent web crawlers', could be invaluable to microbiologists for such analyses. Many business intelligence applications are already exploiting web robots or autonomous programs that methodically traverse the Internet by following hypertext links and then analyse the results to gain knowledge that enables fulfilment of new tasks. Today, microbiologists often spend more time looking at nucleic acid sequences than at colonies on Petri dishes. Therefore, it seems appropriate to consider robots that could combine the analysis of publicly available genomes of pathogens with the published body of knowledge wide analysis of infectious diseases. Figure 1 illustrates a process flow and methods that might be employed by web robots designed to become intelligent assistants to microbiologists.

### Searching for online services fragmented by pathogen and application

While there has indeed been rapid growth in web portals and specialized datasets dedicated to the storage and molecular analysis of individual pathogens or microbial genera, we still lack comprehensive web-accessible resources that organise, analyse and predict host-pathogen interactions. Large databases with genome annotation and visualisation tools such as the European Bioinformatics Institute (EBI), the Integrated Microbial Genomes (IMG) system and Comprehensive Microbial Resource (CMR) have been developed to allow microbial genomes to be compared and examined from an evolutionary point of view [1-3]. Other services include online platforms for comparative genomic analysis of multiple sequences obtained from metagenomic experiments using the 'next generation sequencing platforms' (e.g., CompostBin [4] and PhyloPythia [5]) or search and reconstruction of metabolic networks for microorganisms like BioCyc, PUMA, Reactome or Meta Shark http://bioinformatics.leeds.ac.uk/shark/[6]. The functional classification tools available on these resources have only a weak capability to mine many-to-many gene-to-term relationships. Recent advances in information retrieval allow to link genes and genomes with relevant publications stored in a public domain [7]. Such 'high-level' analyses are computationally expensive and often require parallel computing on distributed computing systems. Encouraging examples from human genome analyses include systems like Galaxy http://galaxy.psu.edu or CLoVR http://clover.igs.umaryland.edu. Locating online resources which are the most suitable for a specific analytic task in hand applied to a set of individual genes or whole genomes has become a challenge. Web robots with their ability for online service discovery are likely to markedly increase the effectiveness of analytic processes.

### Gaining knowledge by integrating structured and unstructured information

A true systems biology approach to interpreting genome-scale data requires the analysis of interactions among the genes, not just the genes in isolation [8]. The integration of data acquisition from online databases with information retrieval from literature text mining is an important step forward. By discovering and quantifying the associations between genes and functions or clinical syndromes, computational robots would capture critical data for translational biomedicine. For example, these intelligent and tireless robots could be programmed to measure the similarity between pre-defined nucleic acid sequences and to mine the literature content to assess the similarity of the context in which gene names and clinical entities appear in literature. As a result of such integration and data analysis, new knowledge about genome structure and function can be gained. The formal representation of this knowledge as a network reveals genome dynamics or patterns of relationships between genes and infectious disease behaviour in multiple dimensions. These knowledge landscapes can be visualised and potential gaps in our knowledge identified. Such network visualisation of associations has been gaining popularity. For example, several text mining engines have focused on the capture of gene-protein entities and the construction of the resulting 'knowledge networks' [8]. We recently presented the first analysis of the infectious disease knowledge space, created by mining for informative associations between genes and diseases [9]. A network representation of multilevel relationships between infectious disease syndromes and pathogens and their genomes was employed to illuminate non-trivial associations that can point out the biological similarities in disease pathogenesis and epidemiology. We also presented a new scalable computational approach that may improve our understanding of host-pathogen relationships and facilitate the discovery and assessment of hidden associations in biomedicine [9].

While there are several well-studied genes that have hundreds of indexed citations in the literature, that degree of functional annotation falls off steeply: almost 80% of Entrez Gene entries had five or fewer linked references in PubMed and almost 50% had zero linked references [10]. The process of annotating gene function typically entails large-scale efforts by the model organism community and genome annotation centres. Inferences regarding the likely function of a molecule are largely based on factors such as homology and interaction partners. However, curated interactions represent less than 1% of the true protein-protein interactions recorded in the Database of Interacting Proteins [8]. While protein interactions are reasonably well characterised experimentally in yeast, protein interactions in prokaryotic organisms remain less understood. The traditional model of centralised curation is not scaling well with the rate of data generation, and complementary approaches based on community intelligence have been advocated [10].

Unstructured knowledge indexed by the National Library of Medicine and made available through PubMed has an annual growth rate of more than 4.8%. Structured knowledge in the form of molecular biology databases has also increased significantly. An annual compendium of molecular biology databases in 2008 contained 1, 078 databases. Platforms like caBIG (Cancer Biomedical Informatics Grid), i2B2 (integrating biology and bedside) and GenGIS have taken on the challenge of the integration of different data types from genomes and associated phenomes in order to allow the study of whole genome associations and the identification of 'hot-spots' for new drugs [11].

### Applying new knowledge to interpret genomes and to address 'big' evolutionary questions

The capacity of web robots to generate new associational knowledge makes them far more powerful than currently used workflow engines, which intend to automate data analysis tasks [12-14]. Translational robots are to be charged with the task of applying genome-phenome-syndrome associations to optimize the profiling of pathogens with epidemic potential. A pathogen profile can be defined as a set of attributes or markers that allow comparison with existing or future databases and linkage of clinical observations and patient outcomes to genome, transcriptome, proteome and metabolome data [15]. Pathogen profiling would allow prognostic categorization of patients and validation of markers that can be used for earlier diagnosis and to predict and monitor response to treatment. These biomarkers, in turn, should also improve efficiency in outbreak investigation and disease monitoring.

The continuing strong growth (with a current rate of about 650 genomes per year) in genomic data is enabling new high-resolution surveillance of organisms as well as assisting the study of 'big' unanswered evolutionary questions. Coupled with the repeated sampling of pathogen populations, these approaches enable the assessment of genome phylodynamics, evolutionary scales and the richness of microbial communities [11,16]. Genome sequences form an essential starting point for genome-wide functional studies aiming to decipher the mechanisms involved in the colonisation of the host and subsequent infection transmission and disease. Comparative analyses of these sequences, such as genome alignments and preliminary annotation of coding sequences, can point to traits associated with macro- and micro-diversity. They refine our understanding of the population biology of microbial species and advance the power of homology inference methods and function predictions. Importantly, they also enable queries in opposite directions from phenome to genome to reveal functions of hypothetical proteins and unknown coding sequences.

The complexity of analysis of pathogen genomes has increased markedly, but there has been an even greater increase in opportunities for an immensely deeper understanding of the causes of disease and of better targeting interventions [17]. However, despite the ever-increasing quantity of papers published on the role of genes in diseases and the growing number of prokaryotic and eukaryotic genomes and individual genes being deposited into public databases, efficient tools to support knowledge discovery remain few and far between. Translational robots are likely to close this gap and to help in the testing of the key assumption that the synthesis of microbial genomic data with clinical and population health data and publicly available evidence actually improves the quality of clinical decision making and public health outcomes.

Science has been lagging behind other industries which have been heavily investing in business intelligence in the last decade, with an 11.2% growth of investment in these technologies since 2006 [18]. Recent initiatives like the RoboEarth project http://www.roboearth.org, aiming to build a parallel World Wide Web for robots where they can share the information and learn from each other, are paving a new road for data analysis. The concept of web robots allows us to address the growing gap between the exponential growth of publicly available data and our capacity to leverage it. Robotic assistants can improve the knowledge utilization, accelerate discovery by searching and challenge the position of observation and experimentation as the most productive means of discovery. Computational data integration and analysis still has a long journey ahead of it, but the light is beginning to shine brightly on its path. We are another step closer to Norbert Wiener's prediction that machines might eventually devalue the brain as emphatically as the first Industrial Revolution did the hand.
